# Eupatilin Inhibits Reactive Oxygen Species Generation via Akt/NF-κB/MAPK Signaling Pathways in Particulate Matter-Exposed Human Bronchial Epithelial Cells

**DOI:** 10.3390/toxics9020038

**Published:** 2021-02-18

**Authors:** Dong Chang Lee, Jeong-Min Oh, Hyunsu Choi, Sung Won Kim, Soo Whan Kim, Byung Guk Kim, Jin Hee Cho, Joohyung Lee, Ji-Sun Kim

**Affiliations:** 1 Department of Otorhinolaryngology Head and Neck Surgery, College of Medicine, The Catholic University of Korea, Seoul 06591, Korea; sayman@hanmail.net (D.C.L.); kswent@catholic.ac.kr (S.W.K.); kshent@catholic.ac.kr (S.W.K.); coolkim@catholic.ac.kr (B.G.K.); entcho@catholic.ac.kr (J.H.C.); fpsljh@gmail.com (J.L.); 2 Clinical Research Institute, Daejeon St. Mary’s Hospital, Daejeon 34943, Korea; 20130097@cmcdj.or.kr (J.-M.O.); peace420@cmcdj.or.kr (H.C.)

**Keywords:** eupatilin, particulate matter, ROS, bronchial epithelial cell

## Abstract

Background: Eupatilin is an active flavon extracted from the Artemisia species and has properties such as antioxidant, anti-inflammatory, and anti-cancer. We examined the effect of eupatilin using fine particulate matter (FPM) and human bronchial epithelial cell line (BEAS-2B) to confirm the potential of eupatilin as a therapeutic agent for respiratory diseases caused by FPM. Methods: Reactive oxygen species (ROS) levels were checked by flow cytometry to identify if FPM and eupatilin affect ROS production. Western blotting was performed to identify the mechanism of action of eupatilin in FPM-exposed BEAS-2B cells. Results: When cells were exposed to FPM above 12.5 μg/mL concentration for 24 h, ROS production increased significantly compared to the control. When eupatilin was added to cells exposed to FPM, the ROS level decreased proportionally with the eupatilin dose. The phosphorylation of Akt, NF-κB p65, and p38 MAPK induced by FPM was significantly reduced by eupatilin, respectively. Conclusion: FPM cause respiratory disease by producing ROS in bronchial epithelial cells. Eupatilin has been shown to inhibit ROS production through altering signaling pathways. The ROS inhibiting property of eupatilin can be exploited in FPM induced respiratory disorders.

## 1. Introduction

There are well-documented serious adverse health effects of air pollution on human health. Particulate matter (PM), which is composed of organic compounds, metals, and dust particles, is known to be the cause of air pollution [1]. PM is classified according to the particle size. PM with a diameter of less than 10 µm is deposited mainly in the upper respiratory tract. Fine PM (FPM), which is less than 2.5 µm or less, enters the body through the bronchi by inhalation. Ultrafine particles (PM0.1) less than 0.1 µm can enter the body through the lungs and eventually travel to all organs. Acute and chronic exposure to PM has been shown in several studies to cause various inflammatory reactions in multiple human organs, especially to have a fatal effect on the respiratory system [2,3,4,5].

Respiratory epithelial cells control immunity in the body through various interactions with cells of the immune system and the production of defensive factors [6,7]. In particular, bronchial epithelial cells are at the forefront of defense against FPM [8]. Bronchial epithelial cells have been used in several studies that have identified the effects of FPM and the effects of therapeutic substances related to it [9,10].

In vitro test results have been reported on the mechanisms by which PM can cause respiratory disease. PM induces oxidative stress, cellular DNA damage, mitochondrial transformation, autophagy, and the production of various inflammatory cytokines in the cells of the respiratory system [11,12,13,14]. Substances that inhibit the mechanism of PM-induced damage can be carefully considered as a template for the development of therapeutic agents for PM-induced respiratory diseases.

Eupatilin (5,7-dihydroxy-3,4,6-trimethoxyflavone) is an active flavone extracted from Artemisia species and has been reported to have antioxidant, anti-inflammatory, anti-cancer, and neuroprotective effects [15,16,17,18]. Based on the literature, we hypothesized that eupatilin could reduce oxidative damage to respiratory cells caused by FPM. The purpose of our study was to confirm the potential of eupatilin as a therapeutic agent for respiratory diseases caused by FPM and to understand its mechanism. We examined reactive oxygen species (ROS) generation by FPM, the effect of eupatilin on it, and its mechanism of action using human bronchial epithelial cell lines (BEAS-2B).

## 2. Materials and Methods

### 2.1. Reagents

Fine atmospheric particulate matter NIST 2786, eupatilin, 5-(and 6)-carboxy-2′,7′-dichlorodihydro-fluorescein diacetate (H2DCFDA), and Collagenase A were obtained from Sigma-Aldrich (St Louis, MO, USA). Antibodies against p38 mitogen-activated protein kinase (MAPK, #9212), phosphorylated p38 MAPK (#4511), Protein Kinase B (Akt, #9272), phosphorylated Akt (#9275), Nuclear factor kappa B (NF-κB) p65 (#8242), phosphorylated p65 (#3036), and Glyceraldehyde-3-Phosphate Dehydrogenase (GAPDH, #5174) were provided by Cell Signaling Technology (Danvers, MA, USA). An inhibitor of Akt (MK-2206) and an inhibitor of NF-κB p65 (BAY11-7082) were purchased from Sigma-Aldrich (St Louis, MO, USA). FPM was suspended in phosphate-buffered saline (PBS). Eupatilin and inhibitors were dissolved in dimethylsulfoxide (DMSO). DMSO alone had no observable effect at concentrations used (data not shown).

### 2.2. Cell Cultures

BEAS-2B cells were purchased from ATCC (Manassas, VA, USA). BEAS-2B cells were cultured in BEGM media with SingleQuot supplements kit additives (Lonza, Walkersville, MD, USA) on a tissue culture flask at an environment of 37 °C and less than 5% CO_2_. BEAS-2B cells were maintained at passages below 20 for all studies.

### 2.3. Cell Viability Assay

The viability of BEAS-2B cells was measured using the WST Assay Kit (DoGen, Seoul, Republic of Korea) to confirm the cytotoxicity of FPM and eupatilin. The WST assay is to measure the amount of viable cells using the function of the cellular mitochondrial dehydrogenase that the tetrazolium salt WST reduces to formazan, and the results are measured with a spectrophotometer. BEAS-2B cells were transferred to each well of a 96-well plate for 24 h (5 × 103 cells/well). After washing twice with PBS, serial concentration of FPM (0–50 μg/mL) and eupatilin (0–100 nM) were added and incubated for 24 h. After adding 10 μL of WST reagent and incubating the cells for an additional 2 h, the absorbance of each well was measured at 450 ± 20 nm in an ELISA reader (Bio-Rad Laboratories, Hercules, CA, USA) to confirm the cell viability (%).

### 2.4. Quantification of Intracellular ROS

BEAS-2B cells were first incubated for 24 h in a 60 mm dish (1 × 10^5^ cells/dish). After washing twice with ice-cold PBS, cells were added with serial concentrations of FPM (0–50 μg/mL) or eupatilin (0–100 nM). The process for detecting intracellular ROS was as follows. BEAS-2B cells were harvested and washed twice with PBS. The cells were then resuspended in Hanks’ Balanced Salt Solution (HBSS) with 10 μM of H2DCFDA. The cell suspension was incubated in the dark at 37 °C for 15 min, then rinsed 3 times with HBSS. The fluorescence intensity of the collected cells was measured using FACS Canto 2 (BD Biosciences, Franklin Lakes, NJ, USA).

### 2.5. Western Blotting

The cells were lysed in RIPA buffer (50 mM Tris-HCl pH 7.2, 150 mM NaCl, 1% NP-40, 0.1% sodium dodecyl sulfate, 0.5% sodium deoxycholate, 1 mM phenylmethanesulfonyl fluoride, 25 mM MgCl_2_) containing a protease inhibitor cocktail (Roche Diagnostics, Mannheim, Germany). To measure the concentration of proteins, we used the bicinchoninic acid kit (Pierce, Rockford, IL, USA). In 10% sodium dodecyl sulfate-polyacrylamide gel, 20 μg of protein was separated by electrophoresis and transferred to a nitrocellulose membrane. The membrane was blocked in TBST (10 mM Tris-Cl, 150 mM NaCl, pH 8.0, 0.05% tween-20) containing 5% non-fat powdered milk. Then, after washing 3 times for 5 min each wash with TBST buffer, the membranes were incubated with primary antibodies (1:1000 dilution) overnight at 4 °C. After that, they were washed 3 times with TBST and incubated with anti-rabbit horseradish peroxidase-conjugated secondary antibodies (1:2000 dilution) for 1 h at room temperature. Labeled proteins were identified using enhanced chemiluminescent detection reagents (GE healthcare, Marlborough, MA, USA), and band intensities were analyzed with Image Lab software (Bio-Rad, Hercules, CA, USA).

### 2.6. Statistical Analyses

Data was analyzed using GraphPad Prism version 5 software (Graph-Pad, San Diego, CA, USA) of all experiments repeated at least 3 times. Statistical significance between means ± standard errors of the mean was investigated using one-way analysis of variance followed by Tukey’s post-test. *p* < 0.05 were considered statistically significant.

## 3. Result

### 3.1. Effects of FPM and Eupatilin on the Viability of BEAS-2B Cells

**Figure 1:**
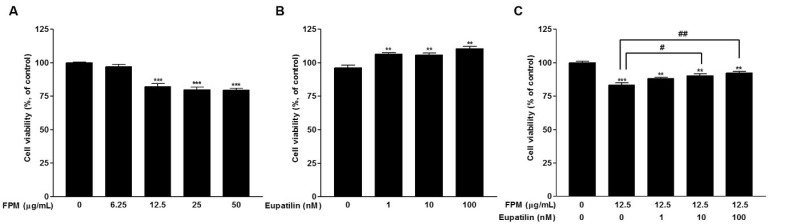
Effects of FPM and eupatilin on BEAS-2B cell viability. (**A**) BEAS-2B cell viability after FPM treatment for 24 h. (**B**) BEAS-2B cell viability after treatment with eupatilin for 24 h. (**C**) BEAS-2B cell viability after treatment with eupatilin and FPM for 24 h. Three independent experiments were conducted. Each value in the graph presented as the mean ± SEM. ** *p* < 0.01 and *** *p* < 0.001 vs. control untreated group; # *p* < 0.05 and ## *p* < 0.01 vs. 12.5 μg/mL FPM. FPM, fine particulate matter; WST, water-soluble tetrazolium salts.

The effect on cell viability was determined by WST assay. BEAS-2B cells were incubated with serial concentrations of eupatilin or FPM for 24 h. Treatment with FPM (>12.5 μg/mL) significantly reduced the viability of BEAS-2B cells (*p* < 0.001) (Figure 1A). On the other hand, eupatilin treatment (>1 nM) significantly increased cell viability (*p* < 0.01). (Figure 1B). The decrease in cell viability due to FPM treatment of 12.5 μg/mL was attenuated by eupatilin, and this effect was statistically significant at concentrations of eupatilin above 10 nM (Figure 1C).

### 3.2. Opposite Effects of FPM and Eupatilin on ROS Generation in BEAS-2B Cells

**Figure 2:**
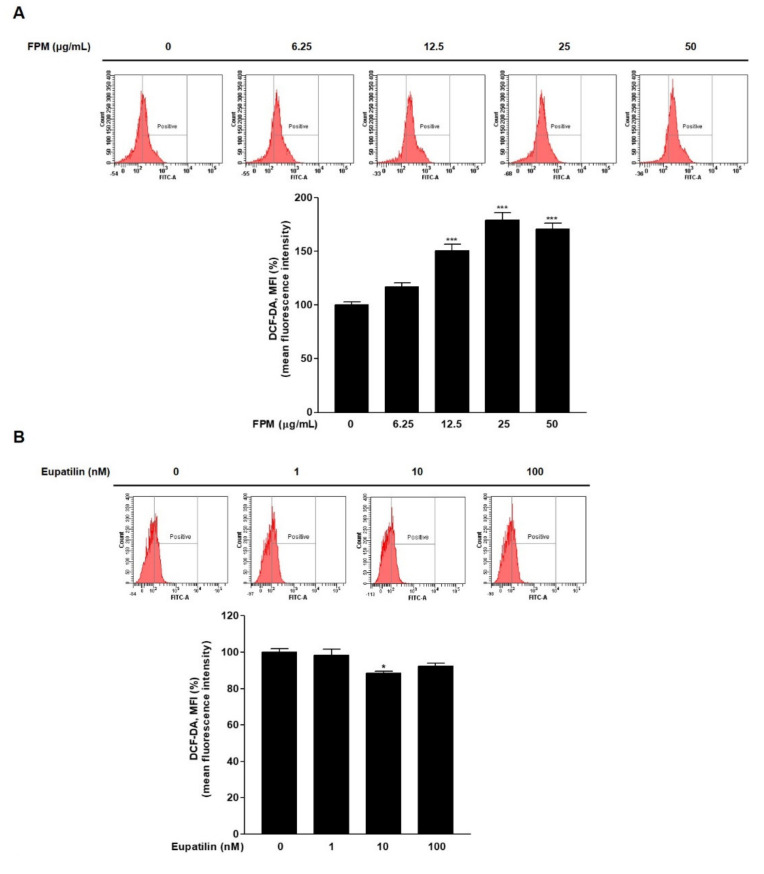

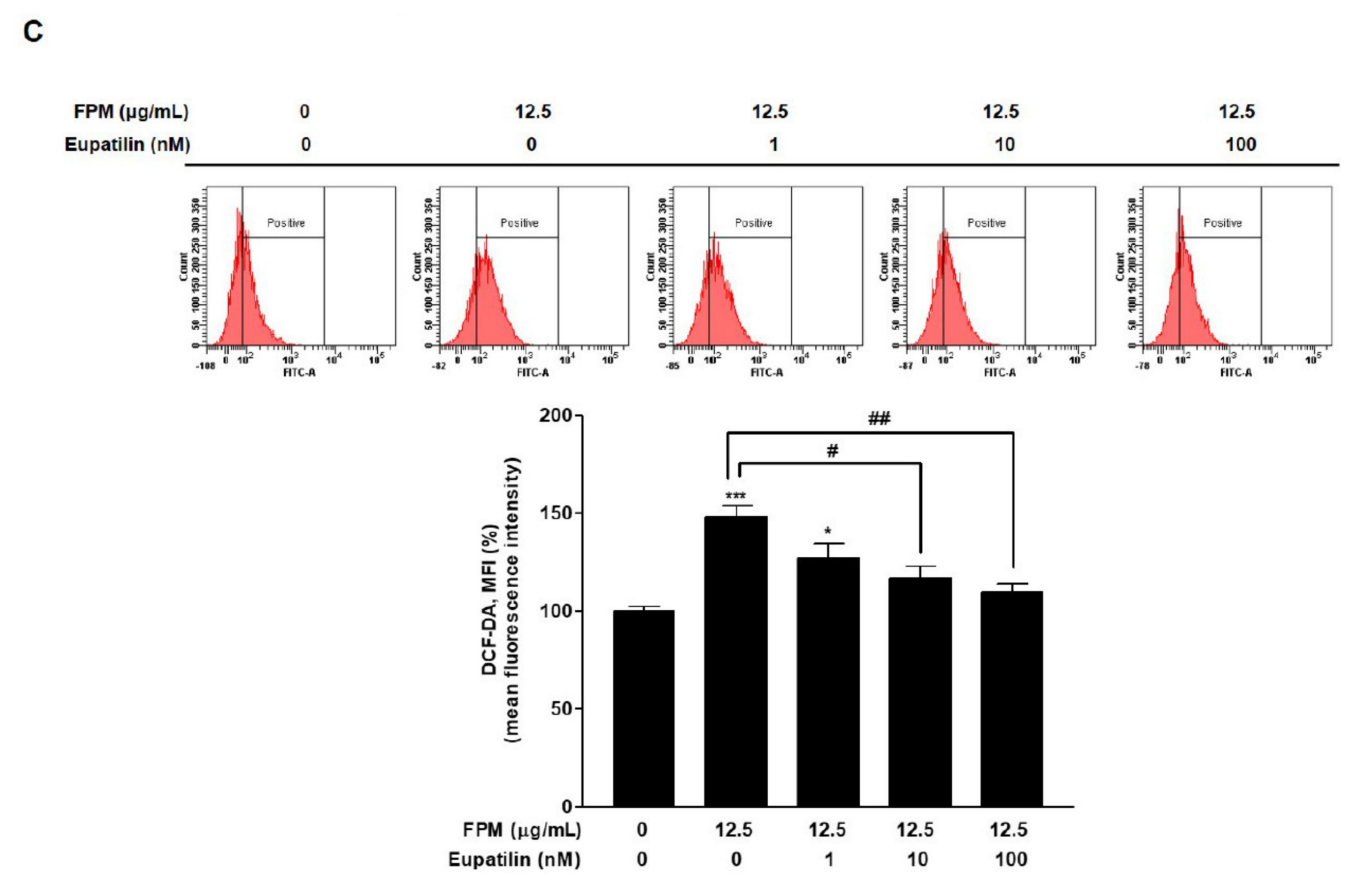
ROS product in BEAS-2B cells treated with FPM or eupatilin as measured by flow cytometry. (**A**) Dose-dependent ROS product in BEAS-2B cells exposed to FPM. (**B**) ROS production depending on the dose of eupatilin in BEAS-2B cells (**C**) Eupatilin decreased FPM induced ROS generation in BEAS-2B cells. Three independent experiments were conducted. Data are presented as the mean ± SEM. * *p* < 0.05, and *** *p* < 0.001 vs. control untreated group; # *p* < 0.05 and ## *p* < 0.01 vs. 12.5 μg/mL of FPM. ROS: reactive oxygen species; FPM: fine particulate matter; SEM: standard error of the mean.

To confirm whether FPM and eupatilin affected ROS generation, ROS levels were confirmed by flow cytometry after staining BEAS-2B cells with DCF-DA. At FPM treatment above 12.5 μg/mL concentration for 24 h, ROS production was significantly increased compared to control (*p* < 0.001) (Figure 2A). ROS production in BEAS-2B cells decreased significantly of exposure to eupatilin (10 nM) compared with the controls (*p* < 0.05) (Figure 2B).

### 3.3. Eupatilin Reduces FPM-Induced ROS Production in BEAS-2B Cells

Flow cytometry was performed to evaluate the effect of eupatilin on FPM-induced ROS production. BEAS-2B cells were pretreated with various dose of eupatilin (0, 1, 10, or 100 nM), and then the cells were incubated in the present or absent of 12.5 μg/mL of FPM for 15 min. ROS levels were significantly increased in the FPM treatment group compared to the control untreated group (*p* < 0.001). In the group treated with FPM and eupatilin, ROS levels decreased proportionally to the dose of eupatilin. (Figure 2C). In particular, eupatilin treatment with a dose of 10 nM or more resulted in a statistically significant reduction compared to treatment with FPM alone.

### 3.4. FPM Induces Phosphorylation of Akt, p65, and p38 in BEAS-2B Cells

**Figure 3:**
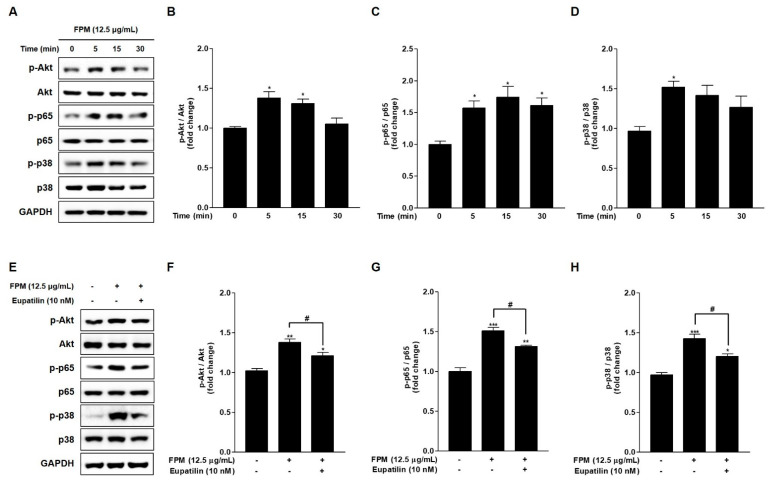
Phosphorylation of Akt, p65, and p38 following the exposure of BEAS-2B cells to FPM (12.5 μg/mL) with or without eupatilin (10 nM). (**A**) Time-dependent phosphorylation of BEAS-2B cells induced by FPM. (**B**) Density ratio of p-Akt to Akt. (**C**) Density ratio of p-p65 to p65. (**D**) Density ratio of p-p38 to p38. (**E**) p-Akt, total Akt, p-p65, total p65, p-p38, and total p38 protein expression levels were determined by Western blot. (**F**) Density ratio of p-Akt to Akt. (**G**) Density ratios of p-p65 to p65. (**H**) Density ratios of p-p38 to p38. Three independent experiments were conducted. Data are presented as the mean ± SEM. * *p* < 0.05, ** *p* < 0.01 and *** *p* < 0.001 vs. control untreated group; # *p* < 0.05 vs. 12.5 μg/mL of FPM. FPM: fine particulate matter; Akt: protein kinase B; GAPDH: Glyceraldehyde-3-Phosphate Dehydrogenase; SEM: standard error of the mean.

To identify the signaling pathways that FPM induced ROS production in BEAS-2B cells, phosphorylation of Akt, NF-κB p65, and p38 MAPK was examined by Western blotting. The expression levels of phosphorylated Akt, NF-κB p65, and p38 MAPK were significantly increased after 5 min exposure to FPM compared to each control group (*p* < 0.05) (Figure 3A–D).

### 3.5. Eupatilin Reduces Phosphorylation of Akt, p65, and p38 in FPM-Exposed BEAS-2B Cells

Western blotting was performed to confirm the mechanism of action of eupatilin in FPM-exposed BEAS-2B cells. The BEAS-2B cells were pretreated with 10 nM of eupatilin or DMSO for 24 h, and then the cells were incubated in the present or absent of 12.5 μg/mL of FPM for 15 min. After incubation, whole cell lysates were subjected to SDS-PAGE and blotted with Akt, p65, and p38 antibodies. The phosphorylation of Akt, NF-κB p65, and p38 MAPK induced by FPM was significantly reduced by eupatilin, respectively (*p* < 0.05) (Figure 3E–H).

### 3.6. Eupatilin as an Inhibitor of the Akt/NF-κB/MAPK Signaling Pathways in FPM-Exposed BEAS-2B Cells

**Figure 4:**
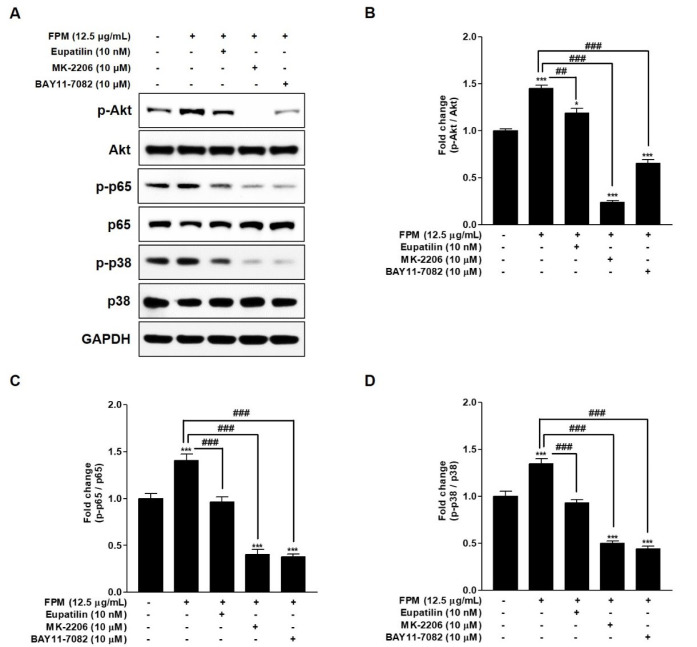
Effects of eupatilin, MK-2206 (Akt inhibitor), and BAY11-7082 (NF-kB inhibitor) on the FPM induced activation of Akt, p65, and p38 in BEAS-2B cells. The BEAS-2B cells were stimulated with FPM (12.5 μg/mL) with or without eupatilin (10 nM), MK-2206 (10 μM), and BAY11-7082 (10 μM of). (**A**) p-Akt, total Akt, p-p65, total p65, p-p38, and total p38 protein expression levels were determined by Western blot. (**B**) Density ratio of p-Akt to Akt. (**C**) Density ratios of p-p65 to p65. (**D**) Density ratios of p-p38 to p38. Three independent experiments were conducted. Each value of the graph represents the mean ± SEM. * *p* < 0.05 and *** *p* < 0.001 vs. 0 μg/mL of FPM and 0 nM of eupatilin; ## *p* < 0.01 and ### *p* < 0.001 vs. 12.5 μg/mL of FPM. FPM: fine particulate matter; Akt: protein kinase B; GAPDH: Glyceraldehyde-3-Phosphate Dehydrogenase; SEM: standard error of the mean; MK-2206: Akt inhibitor; BAY11-7082: p65 inhibitor.

Western blotting was performed by additional treatment with inhibitors of specific signals to confirm the relationship between Akt, p65, and p38, which are involved in the action of eupatilin. The BEAS-2B cells were pretreated with 10nM of eupatilin, 10 μM of MK-2206, and 10 μM of BAY11-7082 or DMSO for 24 h, and then the cells were incubated in the present or absent of 12.5 μg/mL of FPM for 15 min. After incubation, whole cell lysates were subjected to SDS-PAGE and blotted with Akt, p65 and p38 antibodies. Phosphorylation of NF-κB p65, p38 MAPK and Akt was inhibited when BEAS-2B cells exposed to FPM were co-cultured with MK-2206, which acts as an Akt inhibitor (Figure 4). This is a statistically significant inhibitory effect when compared to the group exposed only to FPM (*p* < 0.001). In BEAS-2B cells exposed to FPM after pretreatment with BAY11-7082 acting as an NF-κB inhibitor, NF-κB p65 as well as Akt and p38 MAPK were inhibited. In FPM-exposed BEAS-2B cells, eupatilin inhibited the activities of Akt, NF-κB p65, and p38 MAPK.

### 3.7. Inhibitory Effect of Eupatilin, MK-2206, and BAY11-7082 on ROS Products in BEAS-2B cells

**Figure 5:**
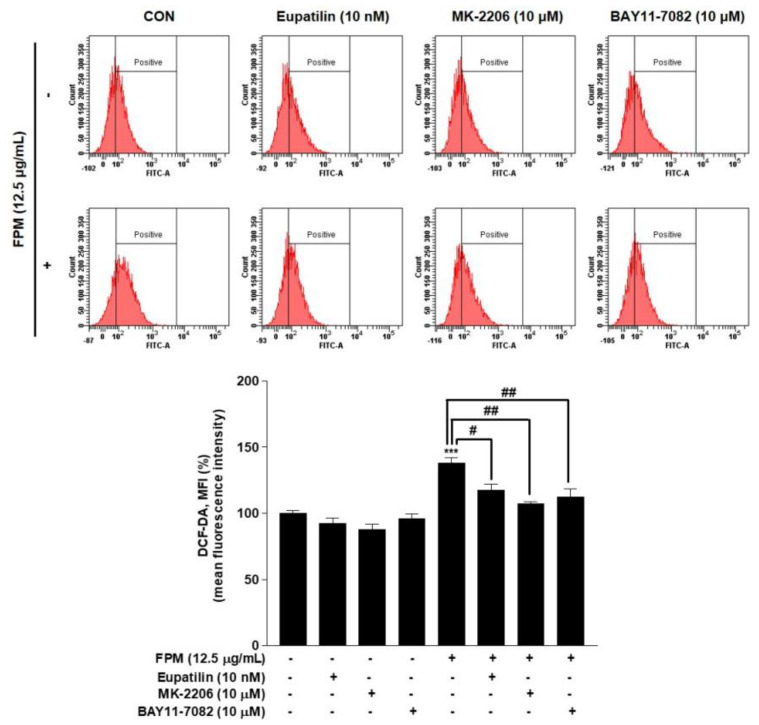
Effects of eupatilin, MK-2206 (Akt inhibitor), and BAY11-7082 (NF-κB inhibitor) on ROS products in BEAS-2B cells. The cells were stimulated with FPM (12.5 μg/mL) with or without eupatilin (10 nM), MK-2206 (10 μM), or BAY11-7082 (10 μM). Three independent experiments were conducted. Data are presented as the mean ± SEM. *** *p* < 0.001 vs. control untreated group; # *p* < 0.05 and ## *p* < 0.01 vs. 12.5 μg/mL FPM. Akt, protein kinase B; MK-2206, Akt inhibitor; BAY11-7082, p65 inhibitor; FPM, fine particulate matter.

Flow cytometry was performed to confirm the effect of eupatilin, MK-2206, and BAY11-7082 on ROS production. BEAS-2B cells were pretreated with eupatilin (10 nM), MK-2206 (10 μM), and BAY11-7082 (10 μM) or DMSO as a control for 24 h, and then the cells were incubated in the present or absent of FPM (12.5 μg/mL) for 15 min. ROS levels were significantly reduced in each of the three groups pretreated with eupatilin or inhibitors compared to the group treated with FPM only (Figure 5).

## 4. Discussion

In our results, FPM significantly produced ROS and induced phosphorylation of Akt, NF-κB p65, and p38 MAPK in human bronchial epithelial cells. Eupatilin significantly inhibited the ROS production and the phosphorylation of Akt, NF-κB p65, and p38 MAPK induced by FPM. MK-2206 as an Akt inhibitor and BAY11-7082 as an NF-κB inhibitor inhibited the phosphorylation of Akt, NF-κB p65, and p38 MAPK induced by FPM. Eupatilin also suppressed these signals activated by FPM.

Long- and short-term exposure of FPM to the respiratory system, which is a major entry channel into the body, has significant adverse health effects. It has been confirmed that it exacerbates chronic obstructive pulmonary disease (COPD) and asthma, and causes acute lung damage, resulting in an increase in the length of hospitalization and years of life loss [19,20,21,22]. Basic research has been conducted to identify the molecular mechanisms and therapeutic drugs for this. BEAS-2B cells have been widely used to describe the molecular mechanisms involved in the respiratory toxicity of FPM. FPM induced ROS production, pro-inflammatory cytokine production, autophagy, and cytotoxicity of immune cells through several signaling pathways in BEAS-2B cells [23,24,25].

Endogenous ROS generated during inflammatory reaction damages epithelial cells by oxidizing DNA, lipid, and protein. This is known as an important damage mechanism for respiratory diseases such as COPD and pulmonary fibrosis [26,27]. In a previous study, we confirmed that PM induces ROS production in human nasal fibroblasts that function immunologically in the upper respiratory tract [28]. In this study, ROS was induced by FPM in bronchial epithelial cells that play an important role in the immune control of the lower respiratory tract.

Eupatilin is known to have anti-inflammatory, antioxidant, and anti-cancer properties. There have been studies on the antioxidant role of eupatilin in epithelial cells of various organs [18,29]. As an example, there was an in vivo experiment confirming that eupatilin inhibited the production of ROS and inflammatory cytokines in acute lung injury induced by LPS [17]. As can be seen from our study results, the generation of ROS induced in bronchial epithelial cells due to FPM is inhibited by eupatilin, suggesting that eupatilin could be considered as a treatment for respiratory diseases caused by FPM.

Akt is a key protein in the Akt signaling pathway that promotes cell growth and survival. Urban PM is known to induce an inflammatory reaction by activating Akt in respiratory epithelial cells [30,31]. In particular, it is known that AKT acts on the generation of oxidative stress caused by FPM in respiratory epithelial cells [32]. Akt pathway induces the NF-κB signaling by stimulating the transactivation domain of p65 [33]. Transcription factor p65 is a subunit of the NF-κB family, an essential transcription factor complex for regulating DNA transcription, cytokine production, and cell survival. Excessive ROS generation activates the inhibitor of NF-κB (IκB) kinase, which degrades IκB and eventually releases transcription factors such as p65 and p50, leading to the production of inflammatory cytokines [34,35]. Several studies have reported that FPM induces inflammatory reactions in respiratory epithelial cells through the NF-κB pathway [36,37]. p38 MAPK is a class of MAPKs that is involved in cell differentiation, apoptosis, and autophagy. This MAPK pathway is activated by environmental stress and inflammatory stimulation, and this process is commonly known to coexist with the NF-κB pathway [38]. ROS phosphorylates p38 and induces apoptotic cell death [39]. In our results, phosphorylation of Akt, p65, and p38 was induced by FPM, and eupatilin inhibited it. For this reason, it was postulated that the Akt, NF-κB, and MAPK signaling pathways acted as the inhibitory mechanisms of eupatilin.

We confirmed that MK-2206, a selective Akt inhibitor, inhibited the phosphorylation of Akt, p65, and p38 induced by FPM. BAY11-7082, as an inhibitor of IκB kinase, inhibits the activation of NF-κB signaling and inhibits the phosphorylation of Akt, an upstream signaling event of NF-κB signaling as well [40]. Likewise, in the present study, BAY11-7082 was found to inhibit the activity of p65 and p38 while inhibiting Akt. However, it is important to be careful in interpreting the results of this study, that it is difficult to determine the signaling cascade by identifying the higher levels of these pathways only with the results of our study.

Eupatilin acts to inhibit phosphorylation of Akt, NF-κB p65, and p38, like MK-2206 and BAY11-7082 in bronchial epithelial cells exposed to FPM. This results of in vitro test does not directly reflect the clinical effects of FPM and eupatilin on the respiratory tract. However, the effect of eupatilin can be applied to the development of new drugs to treat various diseases caused by FPM. There is still insufficient research on the pharmacological structure indicating the effect of eupatilin, and studies on the dose for therapeutic effect and the various toxicities that may appear due to this are needed in the future. Biochemical approaches and in vivo testing are essential for the clinical usefulness of eupatilin.

## 5. Conclusions

FPM cause respiratory disease by producing ROS in bronchial epithelial cells. The Akt/NF-κB/MAPK signaling pathway is suggested to be a pathogenic mechanism for this process, and eupatilin could inhibit ROS production by inhibiting this mechanism. This is a meaningful result confirming the potential of eupatilin as a therapeutic agent at a time when research on respiratory diseases caused by FPM is pouring out. Further experiments on the chemical properties of eupatilin and in vivo studies will bring us one step closer to the development of treatments for respiratory diseases caused by FPM.
